# Genotyping-by-Sequencing (GBS) Revealed Molecular Genetic Diversity of Iranian Wheat Landraces and Cultivars

**DOI:** 10.3389/fpls.2017.01293

**Published:** 2017-08-29

**Authors:** Hadi Alipour, Mohammad R. Bihamta, Valiollah Mohammadi, Seyed A. Peyghambari, Guihua Bai, Guorong Zhang

**Affiliations:** ^1^Department of Plant Breeding and Biotechnology, Faculty of Agriculture, Urmia University Urmia, Iran; ^2^Department of Agronomy and Plant Breeding, Faculty of Agriculture, University of Tehran Karaj, Iran; ^3^Agronomy Department, Kansas State University, Manhattan KS, United States; ^4^Hard Winter Wheat Genetics Research Unit, United States Department of Agriculture – Agricultural Research Service, Manhattan KS, United States

**Keywords:** Iranian wheat landraces, genetic diversity, genotyping-by-sequencing, single nucleotide polymorphism, population structure

## Abstract

**Background:** Genetic diversity is an essential resource for breeders to improve new cultivars with desirable characteristics. Recently, genotyping-by-sequencing (GBS), a next-generation sequencing (NGS) technology that can simplify complex genomes, has now be used as a high-throughput and cost-effective molecular tool for routine breeding and screening in many crop species, including the species with a large genome.

**Results:** We genotyped a diversity panel of 369 Iranian hexaploid wheat accessions including 270 landraces collected between 1931 and 1968 in different climate zones and 99 cultivars released between 1942 to 2014 using 16,506 GBS-based single nucleotide polymorphism (GBS-SNP) markers. The B genome had the highest number of mapped SNPs while the D genome had the lowest on both the Chinese Spring and W7984 references. Structure and cluster analyses divided the panel into three groups with two landrace groups and one cultivar group, suggesting a high differentiation between landraces and cultivars and between landraces. The cultivar group can be further divided into four subgroups with one subgroup was mostly derived from Iranian ancestor(s). Similarly, landrace groups can be further divided based on years of collection and climate zones where the accessions were collected. Molecular analysis of variance indicated that the genetic variation was larger between groups than within group.

**Conclusion:** Obvious genetic diversity in Iranian wheat was revealed by analysis of GBS-SNPs and thus breeders can select genetically distant parents for crossing in breeding. The diverse Iranian landraces provide rich genetic sources of tolerance to biotic and abiotic stresses, and they can be useful resources for the improvement of wheat production in Iran and other countries.

## Introduction

Wheat (*Triticum aestivum* L.) is a staple food crop that feeds about 30% of the world population and provides over 20% of the calories consumed by humans (FAO, 2015). Due to a rapidly growing world population and climate changes, breeders and farmers are facing the challenge of increasing wheat production up to 70% by 2050 to meet future demands (FAO, 2009; Ray et al., 2013; Marcussen et al., 2014), which needs a 2.4% of yield increase yearly. However, the current global average rate of crop yield increase is only 0.9% per year, which is far slower than the desired rate (Ray et al., 2013).

Wheat grain yield can be increased by improvement of both crop management practices and genetic improvement of cultivars for high yield potential (Sener et al., 2009). Genetic diversity is the foundation for such genetic improvement (Nielsen et al., 2014; Govindaraj et al., 2015). Iran is part of the wheat center of origin, and Iranian wheat landraces are important genetic resources for new alleles or genes to be used in breeding for new cultivars (Ciaffi et al., 1992). Wheat landraces are distinct and locally adapted accessions collected and grown by farmers may have a high level of tolerance to biotic and abiotic stresses hence, they are able to provide higher sustainable yields under low input agricultural conditions (Zeven, 1998; Skovmand et al., 2002). For example, Iranian landraces PI 1377397 (Toit, 1989) and PI 626580 (Valdez et al., 2012) have been used as the sources of resistance to Russian wheat aphid [*Diuraphis noxia* (Kurdjumov)]. ‘Turkey Red,’ a hard red winter wheat from Turkey, has been the foundation for hard winter wheat cultivars in the United States due to its cold tolerance (Olmstead and Rhode, 2002).

After the green revolution in the mid-20th century (Borlaug, 1968), wheat landraces have been widely replaced with modern semi-dwarf cultivars, which significantly narrowed genetic diversity. Although artificial selection performed during domestication and breeding processes has increased the frequency of favorable alleles controlling yield and input responses, some other desirable alleles such as biotic and abiotic stress resistance have been removed from breeding populations and cultivars. Therefore, genetic diversity of bread wheat is diminishing in breeding programs (Sofalian et al., 2008). Conservation of landraces and their wild relatives becomes a critical measure to avoid genetic erosion and meet future need of wheat yield increase (Chen et al., 1994).

Molecular markers have been widely used to study the population structure and genetic diversity of germplasm collections (Huang et al., 2002; Dreisigacker et al., 2005; Hao et al., 2006, 2008; Cavanagh et al., 2013). Single nucleotide polymorphisms (SNPs) are the most abundant type of sequence variations in plant genomes (Batley and Edwards, 2007). They are suitable for analysis of genetic variation, population structure, marker-trait association, genomic selection, QTL mapping, map-based cloning, and other plant breeding applications that need large number of markers to cover entire genomes (Kumar et al., 2012). High-throughput SNP arrays are available, but the high cost per sample limits their application in breeding. Recently, next-generation sequencing (NGS) provides a high-throughput and cost-effective molecular tool for breeding and has been widely used to speed up breeding processes (Poland and Rife, 2012; Edae et al., 2014). Rapid advances in NGS technology have driven the costs of DNA sequencing down to the point that genotyping-by-sequencing (GBS) can now be used for routine breeding screening in any crops (Elshire et al., 2011). GBS can be used for marker discovery and genotyping simultaneously and many samples can be multiplexed to reduce cost per sample (He et al., 2014). GBS uses restriction enzyme digestion to reduce the complexity of genomes, which makes it possible to analyze plant species with large and complex genomes such as wheat, and the low cost per sample makes it feasible for breeders to use it as a routine tool to aid breeding selection. An improved GBS protocol (Poland et al., 2012b) that uses two-enzyme (*PstI*/*MspI*) digestion provides a greater degree of complexity reduction and more uniform libraries for sequencing than the original single enzyme protocol (Elshire et al., 2011). The improved GBS protocol has been successfully used in cereal crops such as barley, wheat and oat (Poland et al., 2012a,b; Poland J.A. et al., 2012; Huang et al., 2014).

Iranian wheat landraces provide a rich source of genetic diversity and carry resistance genes to many different biotic stresses such as bunt diseases (Bonman et al., 2015), Russian wheat aphid (Ehdaie and Baker, 1999; Valdez et al., 2012; Bonman et al., 2015), leaf and stripe rusts (Kertho et al., 2015), stem rust (Rouse et al., 2011; Newcomb et al., 2013), and abiotic stresses such as salinity (Jafari-Shabestari et al., 1995), drought and heat (Ehdaie et al., 1988). To date, most of the Iranian germplasm lines have not been characterized and used in modern plant breeding (Hoisington et al., 1999). These germplasm lines not only provide new sources of resistance to biotic and abiotic stresses, but also can enhance the biodiversity of the breeding materials (Huang et al., 2010). Therefore, assessing genetic variation and differentiation of Iranian wheat landraces and cultivars will facilitate the effective use of these valuable genetic resources in future breeding to broaden the genetic diversity of Iranian breeding materials and identify novel alleles that could be used by geneticists and breeders in Iran and other countries. To the best of our knowledge, the present study is the first to directly compare the population diversity of Iranian wheat landraces to a representative pool of Iranian wheat cultivars using SNP markers.

## Materials and Methods

### Genetic Materials

A set of 369 Iranian hexaploid wheat accessions (**Supplementary Table S1**) were used in this study. They included 270 landraces collected between 1931 and 1968 in different climate zones (Supplementary Figure S1) and 99 cultivars released between 1942 and 2014. These were kindly provided by the University of Tehran (UT) and Seed and Plant Improvement Institute (SPII), Karaj, Iran, United States Department of Agriculture-Agricultural Research Service (USDA-ARS)-National Plant Germplasm System, United States, and International Center for the Improvement of Maize and Wheat (CIMMYT), Mexico.

### GBS Library Preparation and Sequencing

Genomic DNA was extracted using a modified cetyltrimethyl ammonium bromide (CTAB) method (Saghai-Maroof et al., 1984) from five 2-weeks-old seedlings. DNA concentration was quantified using the Quant-iT^TM^ PicoGreen^®^ dsDNA Assay (Life Technologies, Inc., Grand Island, NY, United States) and normalized to 20 ng/μl for library construction.

The GBS libraries were constructed following Poland et al. (2012a). In brief, genomic DNA was digested using the restriction enzymes *Pst*I and *Msp*I (New England BioLabs, Inc., Ipswich, MA, United States), and barcoded adapters were ligated to each DNA samples using T4 ligase (New England BioLabs, Inc.). All the ligated products from each plate were pooled and cleaned up using the QIAquick PCR Purification Kit (Qiagen, Inc., Valencia, CA, United States). Primers complementary to both adaptors were used for PCR. The PCR products were then cleaned up again using the QIAquick PCR Purification Kit and quantified using Bioanalyzer 7500 Agilent DNA Chip (Agilent Technologies, Inc.). After size-selection for 250–300 bp fragments in an E-gel system (Life Technologies, Inc.), the concentration of each library was estimated by the Qubit 2.0 fluorometer using Qubit dsDNA HS Assay Kit (Life Technologies, Inc.). The size-selected library was sequenced on an Ion Proton sequencer (Life Technologies, Inc.).

Sequence reads were first trimmed to 64 bp, and identical reads were grouped. Then, unique sequence tags were assigned to the sequence groups. The unique tags were aligned internally allowing mismatches of up to 3 bp to identify SNPs within the tags. SNP calling pipeline was employed as described by Poland et al. (2012b). This pipeline is implemented in TASSEL 3 and was functionally identical to UNEAK to the point of developing a binary presence/absence matrix for each tag across multiple lines. To identify putative SNPs, tags were internally aligned allowing up to 3 bp mismatch in a 64 bp tag. From aligned tags, SNP alleles were identified and the number of lines in the population with each respective tag was tallied in a 2 × 2 table, counting the number of lines with one or the other tag, both, or neither. A Fisher Exact Test was then used to determine if the two alleles were independent, as would be expected for a single locus, bi-allelic SNP in a population of inbred lines. If the null hypothesis of independence for the putative SNP was rejected (*p* < 0.001), we assumed that the tags were allelic in the population (and, therefore, that the putative SNP was a true single locus, bi-allelic SNP). A significance threshold of *p* < 0.001 was selected for the size of population, based on previous work testing false discovery rates in duplicate samples.

Single nucleotide polymorphism calling was conducted using the UNEAK (Universal Network Enabled Analysis Kit) GBS pipeline (Lu et al., 2013), which is part of the TASSEL 4.0 bioinformatics analysis package (Bradbury et al., 2007). Reads with the low-quality score (<15) were removed, and SNPs with heterozygotes < 10%, a minor allele frequency > 1%, and missing data < 20% were used for further analysis. BLASTn analysis was carried out to align sequence reads to the flow-sorted Chinese Spring survey sequence (CSSS) (International Wheat Genome Sequencing [IWGS], 2014) and the Popseq W7984 sequence reference (Chapman et al., 2015) and their location on the genetic map (cM) were predicted through the comparison with GenomeZipper, 90K consensus map and POPSEQ (International Wheat Genome Sequencing [IWGS], 2014).

### Data Analysis

Genetic diversity analysis was performed using DARwin version 6.010 software (Perrier and Jacquemoud-Collet, 2006) and the Jaccard index. The diversity tree was built using WPGMA and Neighbor-Joining algorithm (Saitou and Nei, 1987) that relaxes the assumption of equal mutation rates over space and time and produces an un-rooted tree. The confidence interval of the genetic relationships among the accessions was determined by performing 1,000 bootstraps, with the results expressed as percentages at the main nodes of each branch. Analysis of molecular variance (AMOVA) was used to partition the genetic variation into inter- and intra-gene pool diversities using Arlequin V3.5 software (Excoffier and Lischer, 2010). For analysis of population structure, a model-based Bayesian cluster analysis was performed using STRUCTURE version 2.3.4 (Pritchard et al., 2000). The structure analysis was run 10 times for each K value (K = 1 to 8) using a burn-in period of 10,000 steps and 10,000 MC steps and an admixture model. All parameters were set to default values recommended by the manufacturer (Pritchard et al., 2010). The probability of best fit into each number of assumed clusters (K) was estimated by an ad hoc statistic ΔK based on the rate of change in the log probability of data between consecutive K values (Evanno et al., 2005).

## Results

**FIGURE 1:**
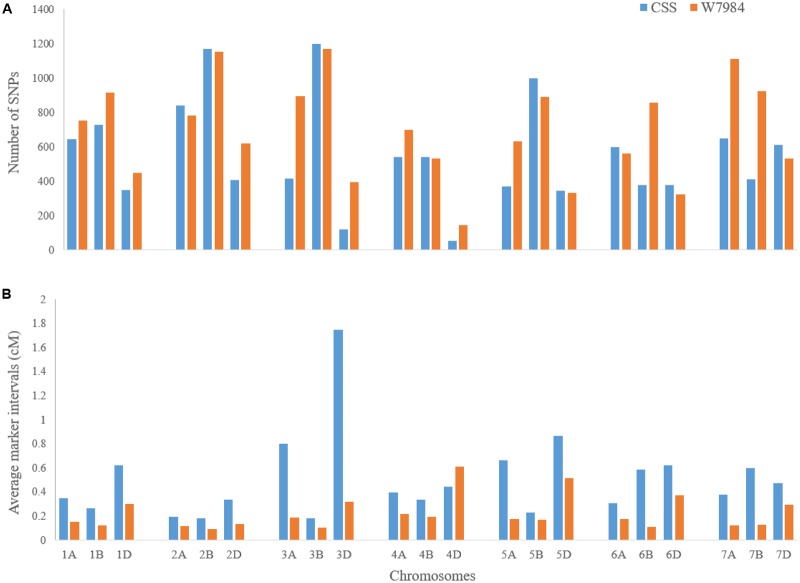
**(A)** Numbers of SNPs and **(B)** average marker intervals (cM) of all 21 wheat chromosomes based on CSSS and W7984 reference assemblies.

**FIGURE 2:**
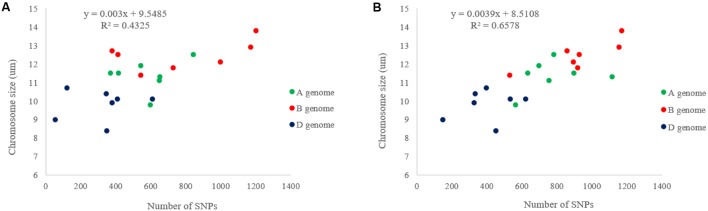
Relationship between numbers of SNPs per chromosome and chromosome physical size estimated in **(A)** CSSS and **(B)** W7984 assemblies.

**FIGURE 3:**
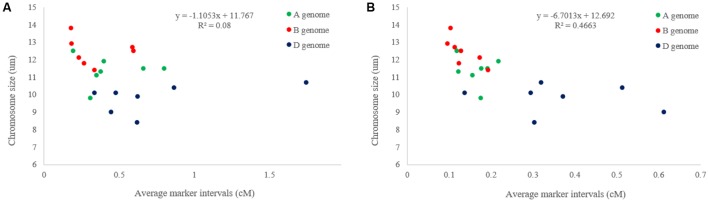
Correlation of average marker intervals (cM) per chromosomes with physical chromosome size estimated by **(A)** CSSS and **(B)** W7984 assemblies.

After eight Ion Proton runs of 369 samples, a total of 566,439,207 reads were identified with 458,363,607 (about 81%) unique reads. A total of 133,039 GBS-SNPs were called after filtering out duplicated reads. Among them, 16,506, 38,824, and 56,560 GBS-SNPs have <20, <50, and <80% missing data, respectively. Only the SNPs with <20% missing data were used to evaluate the genetic diversity of the diversity panel. In the BLASTn analysis, a total of 11,758 (∼71.24%) and 14,697 (∼89.04%) SNPs were aligned to CSSS and W7984 reference genomes, respectively. The highest numbers of SNPs were mapped on the B-genome and the lowest on the D-genome in both CSSS and W7984 references. The highest values of transition-type SNPs were identified on the B genome with 3,465 and 4,107 SNPs while the lowest were on the D genome with 1,397 and 1,721 SNPs on the CSSS and W7984 references, respectively (**Table 1**). A similar chromosome distribution pattern was observed for transversion-type SNPs. More transition-type SNPs (63.63%) were observed than transversion-type SNPs (36.37%) with a transition/transversion (Ts/Tv) SNP ratio of 1.75 (10,503/6,003) over all three genomes. However, the ratio was significantly higher in the A genome than those in the B or D genomes. As expected, more A/G and C/T transitions ware observed than G/A and T/C transitions. On the other hand, more C/G, A/C, G/T, and A/T transversions were observed than G/C, C/A, T/G, and T/A transversions. The SNP numbers in the most of the chromosomes of W7984 assembly were higher than those of CSSS, with only a few exceptions (**Figure 1A**). Similarly, the average marker density was lower in D-genome than in other two genomes of both references (**Figure 1B**). The maximum marker density per chromosome of W7984 assembly (≈10.4 SNP/cM) was much higher than that from CSSS assembly (5.5 SNP/cM). The largest marker intervals were observed for chromosome 3D (∼1.77 cM) of CSSS and 4D (∼0.62 cM) of W7984 assembly. Highly significant correlation was observed between the number of SNPs per chromosome and their physical size for both CSSS (**Figure 2A**) and W7984 (**Figure 2B**), though this correlation was much higher for SNPs called using W7984 reference than CSS reference. The negative correlation between average marker intervals and chromosome sizes was significant for SNPs called using W7984 reference (**Figure 3B**), but not significant for SNPs called using the CSSS reference (**Figure 3A**).

**Table 1:** A summary of single nucleotide substitutions identified in the three homoeologous wheat genomes based on CSSS and W7984 reference assemblies.

	CSSS reference (International Wheat Genome Sequencing [IWGS], 2014)				W7984 reference (Chapman et al., 2015)				
			
Genome	A	B	D	Unassigned	A	B	D	Unassigned	Total
No. of SNPs	4067	5425	2266	4748	5442	6449	2806	1809	16506
Chromosome size (Mbp)	5727	6274	4937	–	5727	6274	4937	–	16938
Marker density (SNP/Mbp)	0.710	0.865	0.459	–	0.950	1.028	0.568	–	0.974
A/G	1213	1564	651	1405	1648	1849	793	543	4833
C/T	1190	1527	582	1263	1576	1808	730	448	4562
G/A	131	203	80	173	175	238	108	66	587
T/C	125	171	84	141	165	212	90	54	521
Transition	2659	3465	1397	2982	3564	4107	1721	1111	10503
Ts %	65.38	63.87	61.65	62.81	65.49	63.68	61.33	61.42	63.63
A/T	195	253	136	223	273	309	150	75	807
A/C	310	411	192	367	410	481	234	155	1280
T/A	26	30	12	27	33	35	15	12	95
T/G	21	45	20	47	33	55	27	18	133
C/A	28	47	11	41	40	57	18	12	127
C/G	492	704	305	637	657	841	382	258	2138
G/T	280	383	162	332	363	457	201	136	1157
G/C	56	87	31	92	69	107	58	32	266
Transversion	1408	1960	869	1766	1878	2342	1085	698	6003
Tv %	34.62	36.13	38.35	37.20	34.51	36.32	38.67	38.59	36.37
Ts/Tv ratio	1.89	1.77	1.61	1.69	1.90	1.75	1.59	1.59	1.75


### Population Structure

**FIGURE 4:**
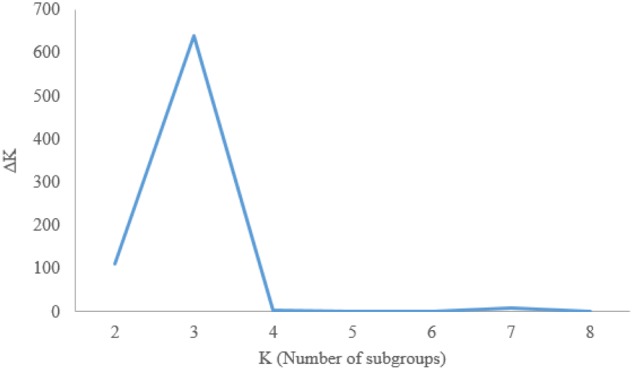
ΔK values calculated for K = 1 to 8 to determine the number of groups in the Iranian wheat landraces and cultivars.

**FIGURE 5:**
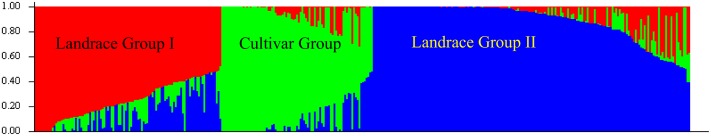
A structure plot of the 369 wheat Iranian landraces and cultivars determined by K = 3 using 16,506 SNPs.

To assess the structure of the Iranian wheat diversity panel, delta K (ΔK) values were used to classify subgroups (K). The largest ΔK was observed at K = 3, suggesting three groups in the panel (**Figures 4**, **5**). Group I contains 104 accessions with 99 landraces and five cultivars, designated as ‘Landrace Group I’; Group II consists of 84 accessions with 80 cultivars and four landraces, designated as ‘Cultivar Group’; and Group III is the largest including 181 accessions with 167 landraces and 14 cultivars, designated as ‘Landrace Group II.’ Most of the cultivars that mixed with landrace group I and II, such as Azar, Biston, Dastjerdi, Deyhim, Homa, Ohadi, Shahi, Sefidak, Shahpasand, and Reyhani, were originally selected from Iranian landraces through continuous selection and purification during the breeding process.

**FIGURE 6:**
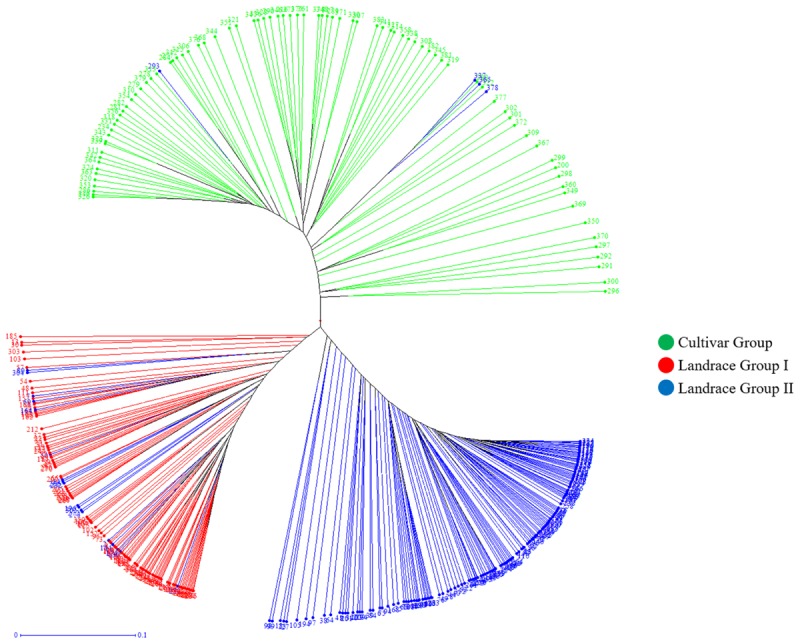
WPGMA clustering dendrogram generated using 16506 SNPs and 369 Iranian hexaploid wheat accessions. Colors reflect groupings derived from structure analysis.

**FIGURE 7:**
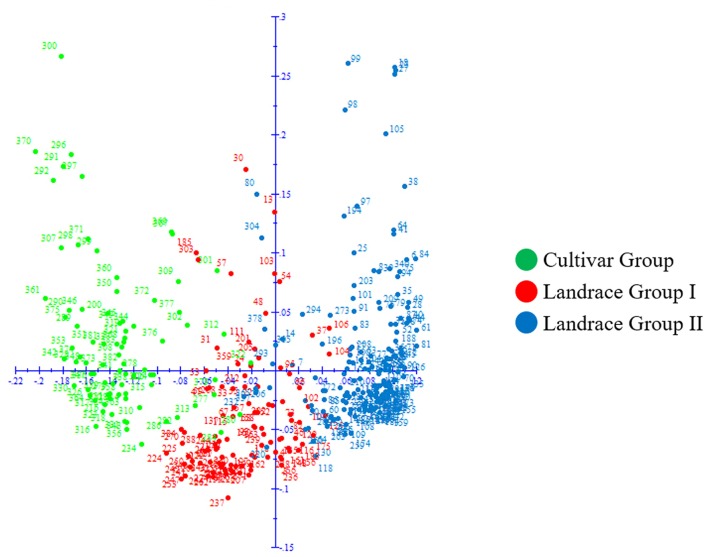
Principal coordinate analysis (PCoA) of 369 Iranian hexaploid wheat accessions based on 16506 SNP markers. Colors reflect groupings derived from structure analysis.

Cluster analysis also classified the panel into three groups that matched with the results from structure analysis (**Figure 6**) and the principal coordinate analysis (PCoA) (**Figure 7**). In PCoA, the first and second coordinate explained 12.03 and 6.20% of the variation, respectively and the Landrace Group I was located between the Landrace Group II and Cultivar Group (**Figure 7**).

**FIGURE 8:**
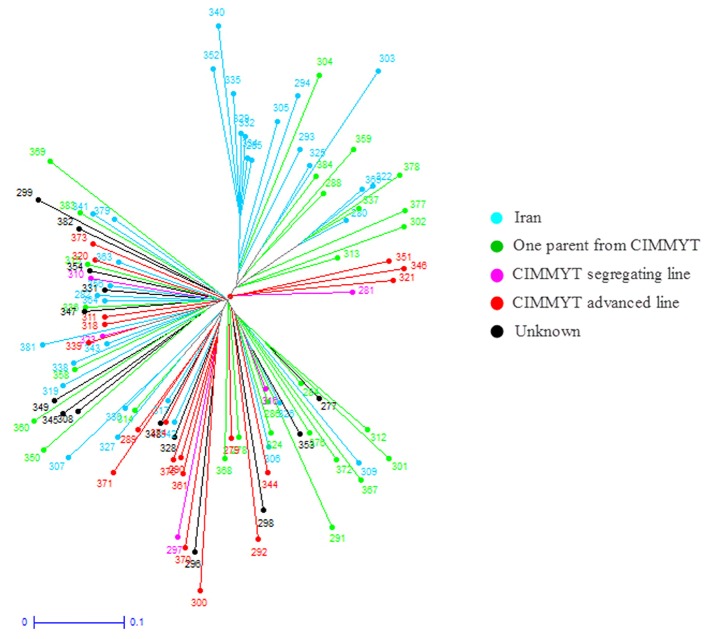
Weighted Neighbor-Joining clustering dendrogram constructed using 16,506 SNPs and 99 Iranian hexaploid wheat cultivars to show their origins as illustrated by different colors.

**FIGURE 9:**
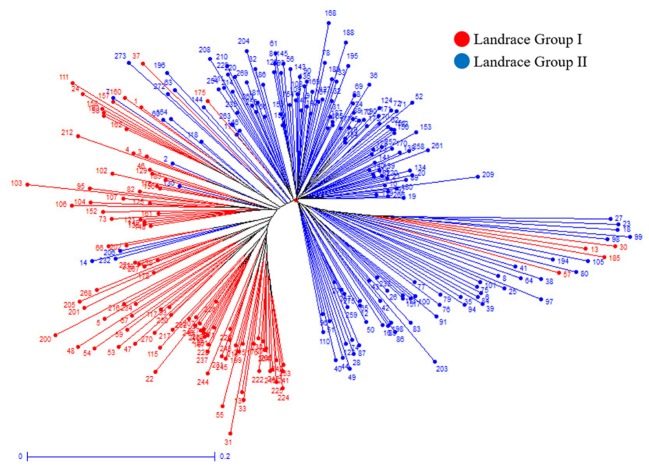
Weighted neighbor-joining clustering dendrogram generated using 16,506 SNPs and 270 Iranian wheat landraces to show the relationship between the groupings from cluster analysis and structure analysis (separation by color).

**FIGURE 10:**
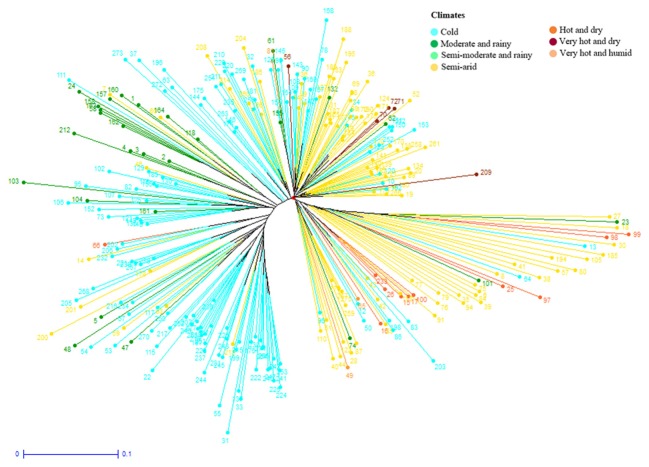
Dendrogram to demonstrate the genetic relationships among 270 Iranian hexaploid wheat landraces based on 16506 SNP markers using Weighted Neighbor-Joining clustering. Climate factors were labeled by colors.

**FIGURE 11:**
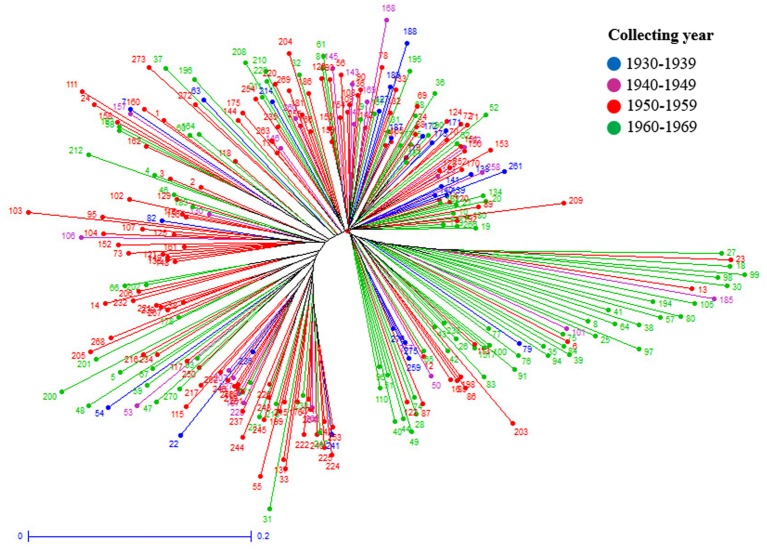
A weighted neighbor-joining clustering dendrogram generated using 16506 SNPs and 270 Iranian hexaploid wheat landraces to show the years of accessions collected.

Further cluster analysis divided the cultivars into four subgroups (**Figure 8**). Most of the cultivars originated from Iran or contain one parent from Iran were separated from those originated from CIMMYT. Cluster analysis on landraces only identified the same two landrace groups as in structure analysis (**Figure 9**). The two landrace groups were grown in distinct climates with Landrace Group I grown under cold or moderate, but rainy climates, and Landrace Group II grown under hot and dry or semiarid climates (**Figure 10**). Also, the two groups can be roughly separated by collection years with Landrace Group I collected before 1960 and Landrace Group II collected after 1960 (**Figure 11**).

### Genetic Diversity

Intra-population genetic diversity analysis revealed that mean observed (N_a_) and effective (N_e_) allele numbers were 1.87 and 1.28, respectively (**Table 2**). The lowest N_a_ was observed in the Cultivar Group (1.779), and the lowest N_e_ was in the Landrace Group II (1.223). The expected heterozygosity (Nei’s gene diversity, H_e_) varied from 0.144 (Landrace Group II) to 0.200 (Landrace Group I). A similar order was observed for Shannon’s diversity index, which varied from 0.240 (Landrace Group II) to 0.320 (Landrace Group I). The lowest private allele number was found in the Cultivar Group (0.012 ± 0.001), whereas both the landrace groups (Landrace Group I and II) showed a higher value of private alleles. Percentage of polymorphic loci per groups ranged from 77.86% (Cultivar Group) to 91.60% (Landrace Group II). Mean marker polymorphism information content (PIC) was low (0.172), ranging from 0.003 to 0.375.

**Table 2:** Genetic variation among three groups identified by structure analysis on a diversity panel of 369 Iranian hexaploid wheat landraces and cultivars.

Diversity index	Landrace group I	Cultivar group	Landrace group II	Average
N_a_	1.915 ± 0.003	1.779 ± 0.005	1.916 ± 0.003	1.870 ± 0.002
N_e_	1.317 ± 0.004	1.310 ± 0.004	1.223 ± 0.003	1.283 ± 0.002
H_e_	0.200 ± 0.002	0.192 ± 0.002	0.144 ± 0.002	0.179 ± 0.001
I	0.320 ± 0.003	0.301 ± 0.003	0.240 ± 0.002	0.287 ± 0.002
Private alleles	0.028 ± 0.002	0.012 ± 0.001	0.031 ± 0.002	0.026 ± 0.002
PPL	91.50	77.86	91.60	86.99 ± 40.57
PIC	Min_PIC = 0.003	Max_PIC = 0.375		Mean_PIC = 0.172


*Values in tables are estimates ± standard error. Na, average number of alleles per locus; Ne, effective number of alleles; He, average expected heterozygosity; I, Shannon diversity index; PPL, percentage of polymorphic loci; PIC, polymorphism information content.*

Analysis of pairwise genetic differentiation and gene flow among three groups (**Table 3**) revealed that the highest value of genetic differentiation was between the Cultivar Group and the Landrace Group II (F_st_ = 0.309), whereas the values were the same between the Landrace Group I to the Cultivar Group and between the two landrace groups. On the other hand, the lowest gene flow was between the Cultivar Group and the Landrace Group I (Nm = 0.559) while the highest was between the two landrace groups (Nm = 1.166).

**Table 3:** Gene flow (Nm, upper right diagonal) and pairwise genetic differentiation (F_st_, down left diagonal) among the two Iranian wheat landrace groups and the cultivar group grouped by structure analysis.

	Landrace group I	Cultivar group	Landrace group II
Landrace group I	–	1.166	1.031
Cultivar group	0.177	–	0.559
Landrace group II	0.195	0.309	–


The AMOVA on the landrace population vs. cultivar population showed a much greater variation within a population (67.36% + 17.08% = 84.44%) than among the populations (15.56%, *p* < 0.001) (**Table 4**). Similar results were obtained using groups derived from structure analysis although slightly higher inter-population variation (22.98%, *p* < 0.001) than within a population was observed. The F_st_ value of 0.16 between landraces and cultivars suggested a substantial degree of differentiation between them while a slightly higher F_st_ value (0.23) among three groups generated by structure analysis also suggests a high differentiation between the two landrace groups.

**Table 4:** Analysis of molecular variance (AMOVA) result from the diversity panel of 369 Iranian hexaploid wheat landraces and cultivars.

	Source of variation	df	MS	Estimated variance	%	^∗^F statistics (Fst)	Probability
Landraces vs. cultivars	Among population	1	6693.47	22.39	15.56	0.16	0.0000
	Among individuals within populations	367	218.02	96.74	67.36		
	Within individuals	369	24.54	24.54	17.08		
	Total of variation	737	129.54	143.63	100.00		
Structure grouping	Among population	2	7765.30	32.66	22.98	0.23	0.0000
	Among individuals within populations	366	194.47	84.96	59.76		
	Within population	369	24.54	24.54	17.26		
	Total of variation	737	129.93	142.17	100.00		


*df, degrees of freedom; MS, mean of squares. ^∗^Index considered as standardized variance of allele frequencies among subdivisions.*

## Discussion

Using the POPSEQ approach, we ordered 11,758 (∼71.24%) and 14,697 (∼89.04%) SNPs to the W7984 and CSSS assemblies, respectively, which were much higher than these reported (22.5–46.3%) in previous studies (Würschum et al., 2013; Shavrukov et al., 2014; Wang et al., 2014). Edae et al. (2015) obtained 33,664 SNPs with up to 80% missing data from W7984 × Opata M85 RIL population out of which, 16,591 (49.3%) and 9709 (28.8%) SNPs were mapped to the W7984 and CSSS reference assemblies, respectively. Distribution of mapped SNPs among the A, B, and D genomes in this study was similar to these in previous reports (Akhunov et al., 2010; Poland et al., 2012a; Würschum et al., 2013; Marcussen et al., 2014; Shavrukov et al., 2014; Edae et al., 2015) with most SNPs mapped to B genome followed by A genome and D genome (Chao et al., 2009; Berkman et al., 2013; Lai, 2015). D genome is the youngest one among the three genomes in wheat evolutionary history. It is likely that older genomes underwent gene duplication and accumulated more mutations that led to sequence polymorphism. Substantial early gene flow could have occurred between *T. aestivum* and its tetraploid progenitor *T. turgidum* (AABB) but not between the hexaploid and *Aegilops tauschii* (DD). This could have resulted in greater sequence diversity in the A and B genomes than in D genome (Talbert et al., 1998; Caldwell et al., 2004; Dvorak et al., 2006; Berkman et al., 2013). In this study, the number of SNPs that mapped to A or B genome were twice as many as those that mapped to the D genome. These results agree with several other studies (Akhunov et al., 2003; Chao et al., 2009, 2010; Wang et al., 2013; Edae et al., 2014; Iehisa et al., 2014) but contradict with some previous reports which indicated that five times more SNPs mapped to A or B genome than to the D genome (Allen et al., 2011, 2013; Cavanagh et al., 2013). This result suggests that Iranian wheat landraces may have relatively higher SNP variation in the D genome than other sources. Higher diversity in the D genome may provide new elite and desirable alleles controlling agriculturally important traits to deal with global climate and environment changes (Trethowan and Mujeeb-Kazi, 2008; Jia et al., 2013).

We observed an average transition/transversion (Ts/Tv) ratio of 1.75 for all three genomes based on both CSSS and W7984 references (1.89 for A genome, 1.76 for B genome and 1.59 on D genome), which reflects the high frequencies of A to G and C to T mutations following methylation. D genome has the smallest Ts/Tv ratio among the three genomes. Higher frequency of transition mutations has been observed in several species including hexaploid wheat (Lorenc et al., 2012; Winfield et al., 2012; Manickavelu et al., 2014) and barley (Turuspekov et al., 2016) with Ts/Tv SNP ratios ranging from 1.59 to 2.12, which agrees with the current study. Transition abundance in many species may result from the mutation of methyl cytosine to uracil and then to thymine (Coulondre et al., 1978). Bread wheat genome is highly methylated due to the two rounds of polyploidy. Therefore, highly repeated sequences and abundant transitions can be considered an ‘evolutionary footprint’ of methylation (Buckler and Holtsford, 1996; Feldman and Levy, 2012; Kalinka et al., 2017). It has been demonstrated that loss of genes occurred more frequently in the A and B genomes than in the D genome (Berkman et al., 2013; Pont et al., 2013). The observed Ts/Tv bias in wheat provides a high level of confidence in SNP prediction accuracy because such a bias was unlikely caused by erroneously called SNPs due to errors in sequencing or mapping (Lorenc et al., 2012).

Chromosome 4D had the least SNPs while chromosome 3B had the highest using both CSSS and W7984 assemblies. On the other hand, a relatively high positive correlation was observed between the number of SNPs mapped to a chromosome and size of the chromosome, which agrees with previous studies.(Poland et al., 2012a; Saintenac et al., 2013; Edae et al., 2015). In addition, other factors such as the time of evolution of a genome also affect the number of SNPs mapped on chromosomes.

Structure analysis placed 369 Iranian wheat accessions into three groups with only 7% of accessions misplaced into an opposite group. The most misplaced accessions were the cultivars that were placed into one of the landrace groups because most of these cultivars were selected from Iranian landraces. Therefore they should belong to landraces. Cluster analysis generated a similar grouping pattern. When pedigrees, geographical regions of cultivation, years of accession released, growth habits and origins of cultivars were analyzed in cluster analysis, we found that accession pedigree is the main factor for separation of the Iranian cultivars. Most cultivars that originated from Iran or with one parent of Iranian wheat were clearly separated from those which originated from CIMMYT, suggesting that Iranian wheat may have a different genetic makeup from the CIMMYT wheat. That may explain why crossing made between Iranian and CIMMYT wheat genotypes produced high yielding cultivars. For instance, Pishgam and Parsi derived from a cross between Iranian and CIMMYT lines are currently the most widely planted cultivars in Iran (Mahfoozi et al., 2009; Amin et al., 2010). For landraces, a high level of genetic diversity was observed among those collected in different years, or from different geographical regions and climate zones. Genetic variation among the landraces collected from north of Iran was higher than the landraces from the south. For example, most of the landraces collected from Gilan and Mazandaran were grouped closely to each other. Landrace collection years can also separate the landraces into two groups based on whether they were collected before and after 1960. This may be attributed to the breeders at University of Tehran who initiated the purification of landraces after 1960. Therefore, genetic variability among the landraces can be affected by the years of collection, anthropogenic impact through dynamic storage practices, diverse needs, end-uses, seed exchange (gene flow) between farmers, and geographic and environmental conditions where wheat grows. It has been documented that genetic diversity of landraces could be related to their adaptability (Cooper et al., 2001) and farmers have played a pivot role in maintaining the genetic diversity (Zeven, 2002).

Genetic diversity for each of the predefined subpopulations was measured using Nei’s and Shannon’s genetic diversity indices, private alleles, the percentage of polymorphic loci and PIC in this study. The Landrace Group I showed higher values of Nei’s and Shannon’s gene diversity indices and the Cultivar Group demonstrated a lower average of private alleles and percentage of polymorphic loci than the two landrace subgroups. This result highlights the strong genetic separation among the groups and specific adaptation of the Iranian cultivars captured in the founder lines of the landraces. But whether the proportion of the genetic differentiation in the subgroups of Iranian landraces and cultivars are due to differentiation during different domestication events or introduction from CIMMYT remains unknown. It is possible that a single domestication event occurred with various hybridizations to endemic genotypes in each region, as has occurred in the domestication of rice (Londo et al., 2006; Huang et al., 2012). Further, the significant genetic differentiation (*p* < 0.001) among the three groups as illustrated by pairwise F_ST_ and AMOVA analyses verified the differentiation of three groups in this panel. However, the Landrace Group I demonstrated a higher gene flow and lower F_ST_ value with the Cultivar Group, suggesting the Landrace Group I might play a bridge role between the Landrace Group II and the Cultivar Group. Most accessions in Landrace Group I originated from cold or moderate and rainy climates, and had been collected before 1960 while those in Landrace Group II were mainly collected from semiarid or hot and dry climates after 1960. Since the landraces were collected from diverse climate regions and altitudes, many of these germplasm lines should be useful sources of genes to be used in breeding to address the challenge of climate change.

## Conclusion

Our studies demonstrated that GBS is a powerful tool for investigating population structure and genetic diversity of wheat landraces and cultivars. In this study, Iranian hexaploid wheat landraces and cultivars collected and released from different years, agro-climatic zones with different growth habits were grouped into three distinct groups. Iranian cultivars can be separated into four major groups with one group was mostly originated from Iranian ancestor(s). Large genetic variation was observed among landraces based on the years of collection, geographical and climate zones. We hope that this genetic diversity will help wheat breeders to select parents for crossing to improve wheat under different climate conditions in Iran and other countries.

## Author Contributions

HA analyzed the data and wrote the paper. GB contributed reagents, Ion Proton sequencing and analysis tools. MB, VM, and SP provided seeds of the genotypes. HA, MB, VM, SP, GB, and GZ contributed to conceive the project and revise the paper. All authors read and approved the final manuscript.

## Conflict of Interest Statement

The authors declare that the research was conducted in the absence of any commercial or financial relationships that could be construed as a potential conflict of interest.

## Supplementary Material

**TABLE S1 |** List and information of the Iranian wheat landraces and cultivars.
