# Supplementary material for: Genotyping-by-Sequencing (GBS) Revealed Molecular Genetic Diversity of Iranian Wheat Landraces and Cultivars
Source: Front Plant Sci. 2017 Aug 29;8:1293. doi: 10.3389/fpls.2017.01293 (PMC5583605; doi:10.3389/fpls.2017.01293)
Supplement: Supplementary file 2 [file Image_1.PDF]

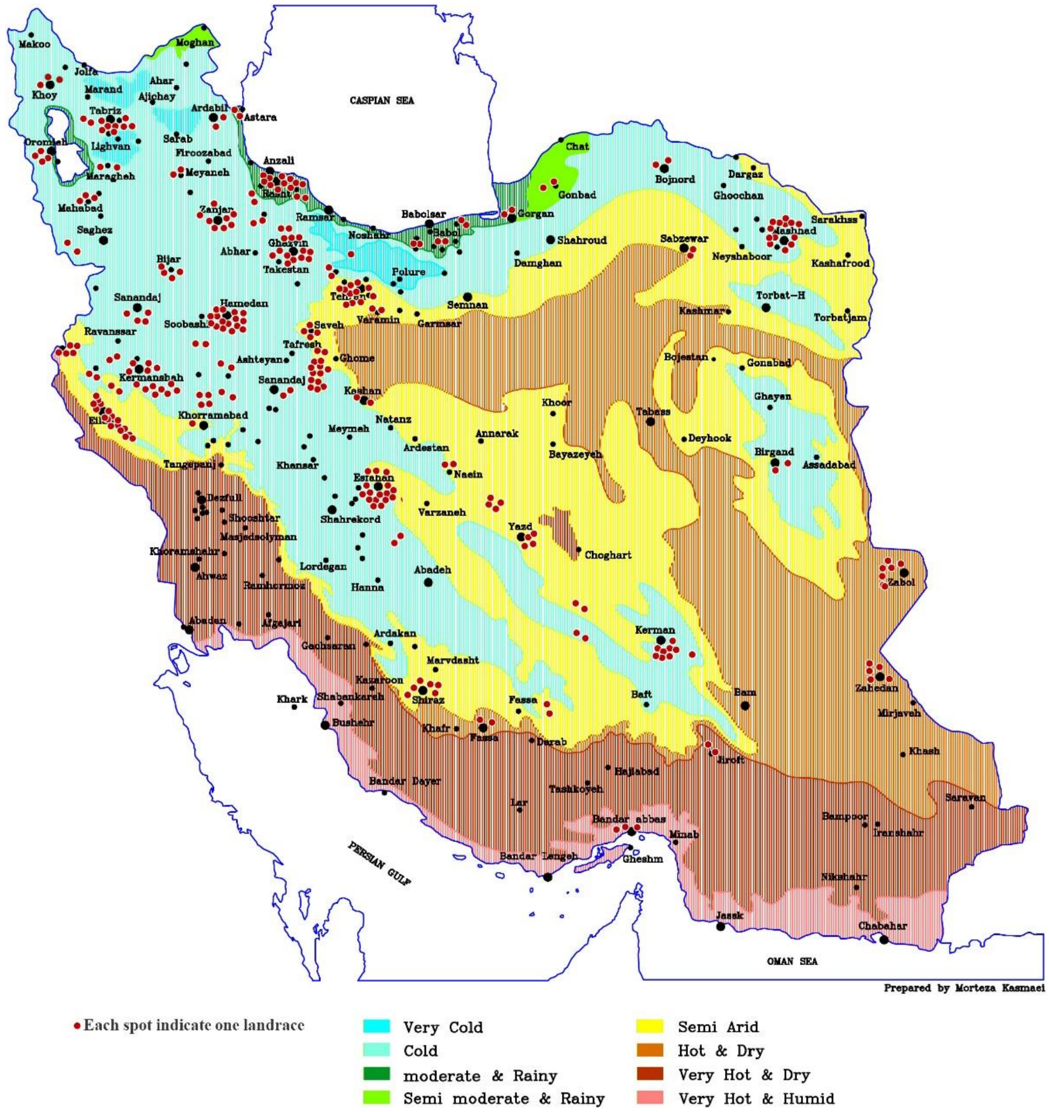

Supplemental figure 1. Geographic distribution of 270 Iranian wheat landraces. Each dot represents one landrace accession.
